# Microbiota variation in *Aedes aegypti* (Diptera:Culicidae): a comparative study of field-caught and laboratory-adapted reared populations

**DOI:** 10.1128/spectrum.03493-25

**Published:** 2026-05-11

**Authors:** Abelgie Sánchez-Amézquita, David Martínez, Omar Cantillo-Barraza, Yurany Granada, Manuel Medina, Luz Helena Patiño, Marina Muñoz-Díaz, Juan David Ramírez

**Affiliations:** 1 Centro de Investigaciones en Microbiología y Biotecnología-UR (CIMBIUR), School of Sciences and Engineering, Universidad del Rosario 537746 , Bogotá, Colombia; 2 Universidad de Antioquia 27983, Medellín, Colombia; 3 Programa para el control de Enfermedades Transmitidas por Vectores de la Secretaria de Salud de Boyacá, Tunja, Colombia; 4 Instituto de Biotecnología-UN (IBUN), Universidad Nacional de Colombia 28021, Bogotá, Colombia; 5 Center for Global Health and Interdisciplinary Research, USF Genomics Program, Department of Global, Environmental and Genomic Health Sciences, College of Public Health, University of South Florida 27117, Tampa, Florida, USA; Connecticut Agricultural Experiment Station, New Haven, Connecticut, USA

**Keywords:** 16S, microbiota, *Aedes aegypti*

## Abstract

The control of *Aedes aegypti*, the main vector of Dengue, Zika, and Chikungunya, remains a global health priority due to its expanding distribution and the absence of specific treatments. The mosquito’s microbiota is increasingly recognized as a factor influencing its biology and vector competence; however, studies comparing wild and laboratory-reared populations are scarce, particularly in Colombia. This study examined the bacterial and microeukaryotic microbiota of *Ae. aegypti* from four municipalities in Boyacá (Puerto Boyacá, Otanche, Moniquirá, and Muzo), alongside laboratory-reared populations maintained for at least five generations and the Rockefeller strain. Microbial profiling was performed using Oxford Nanopore sequencing of 16S and 18S rRNA genes. Results indicated no significant differences in alpha diversity, but beta diversity varied among groups; laboratory-reared mosquitoes showed a higher abundance of *Pseudomonas aeruginosa*, suggesting environmental selection. Despite these variations, a shared microbiota was identified among all groups, including *Cutibacterium acnes*, *Dechloromonas hortensis*, *P. aeruginosa*, *Caldimonas brevitalea*, *Acinetobacter soli*, *Chryseobacterium indologenes*, and *Pseudomonas putida*, which hypothetically suggests the possibility of transstadial transmission. Importantly, this study reports for the first time the presence of the parasite *Ascogregarina culicis* in *Ae. aegypti* from Colombia, a potential modulator of arbovirus transmission that warrants further attention in future studies. Overall, this work provides the first comparative profile of field-collected, laboratory-adapted, and Rockefeller strain populations of *Ae. aegypti* from Boyacá, highlighting the influence of environmental conditions on microbial composition. The bacterial repertoire identified offers candidate symbionts for future functional studies and for the development of new vector-control strategies.

**IMPORTANCE:**

We report changes in the bacterial and microeukaryotic microbiota of *Aedes aegypti* collected in the field and subsequently adapted to laboratory conditions for at least five generations. Our study identified shifts in bacterial beta diversity (principal coordinates analysis, ANOSIM, permutational multivariate analysis of variance; *P* = 0.003), confirming that the rearing environment influences microbial composition. In addition, our findings suggest that certain bacterial microorganisms were associated with both field-collected and laboratory-adapted insects (shared microbiota), which may be relevant to mosquito biology. This study expands our understanding of microbial changes associated with rearing conditions, highlighting their importance for the development of future vector-control strategies.

## INTRODUCTION

*Aedes aegypti* is present in at least 167 countries across all continents except Antarctica, showing strong adaptation to a wide range of environments (1). It is the primary vector of arboviruses such as Dengue (DENV), Zika (ZIKV), Yellow Fever (YFV), and Chikungunya (CHIKV) (2, 3). Its efficiency as a vector depends on the ability of arboviruses to replicate and disseminate without causing significant mortality in the mosquito, thus acting as an exponential biological amplifier (3–5). The rapid spread of DENV in the Americas since the 1980s and of CHIKV in the Caribbean after 2013 illustrates its potential for urban transmission (6). In Colombia, *Ae. aegypti* is currently present in 73.3% of municipalities below 2,200 meters above sea level (masl) (823/1,123) (7), and climate change is expected to facilitate its expansion above 3,000 masl, potentially exposing nearly 30 million people to risk (8).

Currently, there are no specific treatments for arboviral diseases, and vaccine availability remains limited; therefore, vector control continues to be the main preventive strategy (9). Although larvicides, ovicides, and insecticides are widely used due to their cost-effectiveness, their indiscriminate application has favored the development of insecticide resistance in *Ae. aegypti* populations in Colombia (10–12). Considering these limitations, the mosquito-associated microbiota has emerged as a promising target for new control strategies, as it influences vector competence and multiple physiological, metabolic, and immune processes (3, 4, 13, 14). Microorganisms contribute to mosquito development, immunity, insecticide resistance, behavior, and reproduction; for example, *Escherichia coli* produces riboflavin essential for molting (15), immune activation occurs through responses such as melanization and the production of antimicrobial peptides (3, 4, 16), and *Wolbachia pipientis* inhibits DENV replication through the production of reactive oxygen species (17). In addition, bacterial taxa such as *Bacillus* and *Pseudomonas* metabolize insecticides (18, 19), whereas volatile organic compounds influence host preference and oviposition, and *Wolbachia* induces cytoplasmic incompatibility (20–24).

Mosquitoes acquire microorganisms through vertical transmission (25), larval ingestion from aquatic habitats (4, 26, 27), and feeding at the adult stage, and the composition of the microbial community is shaped by geography, diet, and environmental conditions (25, 26, 28). Although a significant microbial loss occurs during pupation (27), stable associations, such as with *Pseudomonas* and *Serratia*, have been documented across generations (29). Laboratory studies reveal that microbial composition is more strongly influenced by rearing conditions than by host genotype (30, 31), although some host-specific associations persist (32). Importantly, adaptation to laboratory environments can reshape microbial composition without reducing diversity (33), highlighting the need to evaluate how these changes affect mosquito biology and vector competence (34). Reports of opportunistic pathogens such as *Candida parapsilosis* in both wild and laboratory-reared mosquitoes further underscore the relevance of microbiota characterization (35).

Therefore, the study of *Ae. aegypti* microbiota in arbovirus-endemic regions such as Colombia is a research priority, as it enables the detection of pathogens and the identification of microbial taxa that are lost, acquired, or maintained under laboratory conditions (30, 31, 36, 37). This is particularly relevant in Colombia, where the insect is present in more than 70% of the territory (7, 38) and is projected to expand into Andean regions such as Boyacá under climate change scenarios (8, 39, 40). However, knowledge gaps remain regarding differences in microbiota between wild and laboratory populations, which hampers the extrapolation of experimental results to natural conditions. To address this, the present study characterized the microbiota of wild *Ae. aegypti* from Boyacá, their laboratory-adapted descendants, and the Rockefeller strain, to evaluate the effects of rearing conditions on microbial composition and to discuss their implications for mosquito biology and vector competence.

## MATERIALS AND METHODS

### Mosquito collection and rearing

#### Rockefeller reference strain

Mosquitoes from the *Ae. aegypti* Rockefeller reference strain were sourced from a colony maintained at the Biology and Control of Infectious Diseases (BCEI) group of the Universidad de Antioquia since 2006. The colony was reared under controlled laboratory conditions at 28°C (±2) and 80% (±5) relative humidity. Adults were provided with a 10% sucrose solution two to three times per week and offered a weekly mouse blood meal. For this study, adult mosquitoes were collected 48 h post-emergence without having received a blood meal. Samples were immediately preserved in RNAlater and transported to the Microbiology Laboratory at Universidad del Rosario, where they were stored at −40°C until processing.

#### Field population sampling

**Fig 1:**
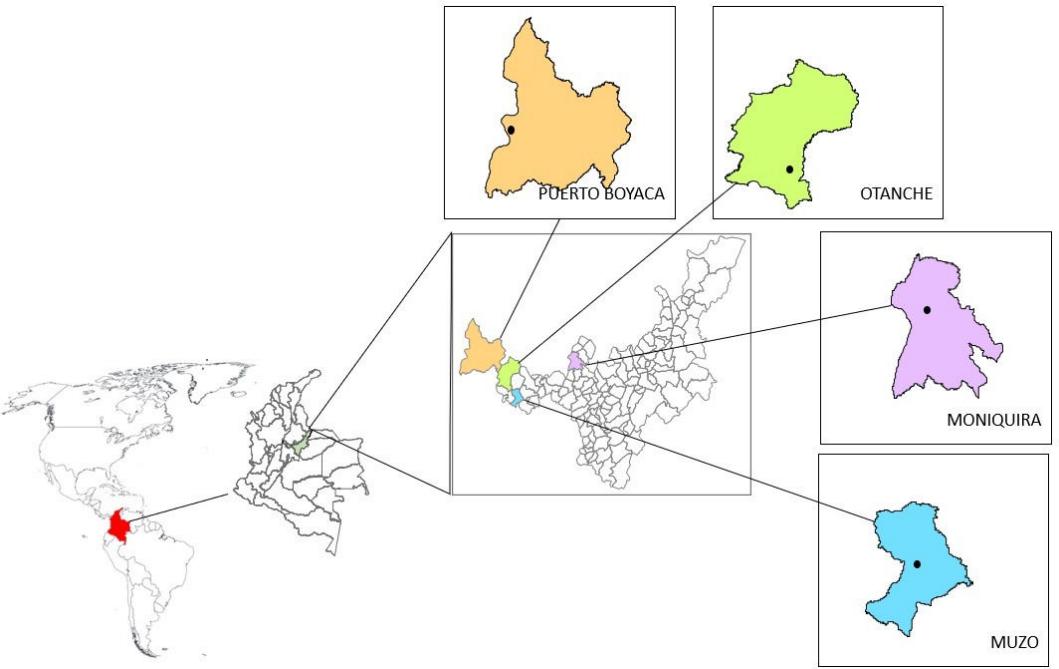
Collection sites of adult and immature mosquitoes in the department of Boyacá, Colombia. Black dots indicate the municipal seats of the sampled municipalities.

Field collections were conducted between February and May 2024 in four municipalities of the Boyacá department, located in the Andes mountain range, an area under YFV outbreak alert and with documented co-circulation of the four DENV serotypes (41): Puerto Boyacá, Otanche, Muzo, and Moniquirá (Fig. 1), in collaboration with personnel from the Boyacá Department of Health. Within each municipality, sampling was conducted in designated neighborhoods (Pueblo Nuevo, El Carmen, Centro, and Coamigos, respectively). Between 15 and 30 households per locality were inspected until approximately 30–40 adult *Aedes* spp. and 200 immature stages had been collected per municipality. Adult mosquitoes were captured indoors (bedrooms, bathrooms, laundry areas, and living rooms) during a single sampling event per locality using entomological nets and oral aspirators. Specimens were immobilized by cold exposure (−4°C), and taxonomic classification was performed using Rueda’s identification keys (42) to distinguish males from females and *Ae. aegypti* from *Aedes albopictus*, with the assistance of entomology staff. Subsequently, mosquitoes were transferred to cryovials containing RNAlater and shipped to the laboratory at Universidad del Rosario, where they were stored at −40°C.

#### Laboratory adaptation of field populations

Immature stages were collected from domestic breeding sites and reared at the BCEI under the same conditions as the Rockefeller strain, with Truchina feeding and, when necessary, water oxygenation. After ≥5 generations in the laboratory, adults were collected 48 h post-emergence without blood feeding and preserved in RNAlater for storage. A total of 223 insects were analyzed. Of these, 32 were collected in the field from Otanche, 30 from Moniquirá, 30 from Puerto Boyacá, and 27 from Muzo. Insects from these populations were subsequently adapted to laboratory conditions: 16 from Otanche for 5 generations, 20 from Moniquirá for 13 generations, 25 from Puerto Boyacá for 5 generations, and 22 from Muzo for 5 generations. In addition, 21 specimens were obtained from the Rockefeller colony.

### Mosquito washing and tissue disruption

To optimize arbovirus detection, individual female *Aedes* mosquitoes from each collection site were processed independently (43). Each specimen was placed individually in a labeled microcentrifuge tube containing 500 µL of DNase/RNase-free water (MP Biomedicals, 2450204). The tube was centrifuged for 3 min at 4,000 rpm in a Spectrafuge 24D microcentrifuge (Labnet). The supernatant (500 µL) was discarded, and this washing step was repeated twice more for a total of three washes per mosquito (31). All procedures were conducted in an ISOCIDE 5237 biosafety cabinet using previously sterilized materials.

Following the washes, each mosquito was transferred to a ZR BashingBead Lysis Tube (S6003-50) containing 300 µL of DNA/RNA Shield buffer. The tubes were homogenized using a TissueLyser II system (Qiagen, Hilden, Germany) at a frequency of 30.0 Hz for 15 min (43). After homogenization, the samples were centrifuged for 2 min at 13,000 rpm, and 200 µL of the resulting supernatant was collected. The samples were then stored at −30°C until further processing.

### Nucleic acid extraction and cDNA synthesis

Total nucleic acids were extracted using the Quick-DNA/RNA Viral MagBead kit (Zymo Research, R2141) on a HAMILTON MICROLAB STAR automated liquid handling system, following the manufacturer’s protocol. The extracted genetic material was stored at −80°C in a Revco freezer (Thermo Fisher Scientific, Waltham, MA, USA). Subsequently, complementary DNA (cDNA) was synthesized from the extracted RNA via reverse transcription using the LunaScript RT SuperMix Kit (New England Biolabs, E3010L). The reaction was performed in a MiniAmp thermal cycler (Thermo Fisher Scientific) with the following thermal profile: 25°C for 2 min, 55°C for 10 min, and 95°C for 1 min. The resulting cDNA was stored at −30°C until use (43).

### Species identification via cytochrome oxidase I (COI) gene PCR

Molecular identification was performed to validate the taxonomic classification. A fragment of the mitochondrial cytochrome c oxidase subunit I gene, a standard molecular barcode for *Aedes* species, was amplified from both field-collected and laboratory-reared specimens, following the protocol described by Martínez et al. (44). Each PCR reaction contained GoTaq Green Master Mix (2×) (Promega, M712B), 10 μmol of each primer LCO1490 (5′-GGTCAAATCATAATAATAAGATATTGG-3′) and HCO2198 (5′-TAAACTTCAGGGTGACCAAAAAATCA-3′), 4.5 µL of nuclease-free water (MP Biomedicals, 2450204), and 1.2 µL of cDNA template. A positive internal control was included in each run.

Amplification was performed in a MiniAmp thermal cycler under the following conditions: an initial polymerase activation at 95°C for 1 min, followed by 40 cycles of denaturation at 94°C for 1 min, annealing at 60°C for 1 min, and extension at 72°C for 1 min, with a final extension step at 72°C for 10 min. The resulting amplicons were separated by electrophoresis on a 2% agarose gel (prepared with 100 mL of 1× TBE buffer and 2 g of agarose) stained with 1 µL of SYBR Safe DNA Gel Stain (Invitrogen, S33102). A band of approximately 650 base pairs (bp) was expected. Amplicons from field-caught insects were subsequently purified with ExoSAP-IT (Applied Biosystems, 78205) and sequenced using the Sanger platform.

### Confirmation of *Aedes* species identity

Species identification was confirmed using the BLASTn tool to perform a local alignment of the COI sequences against the NCBI database, with default parameters. Alignments were considered a positive match for *Ae. aegypti* if they met the criteria of an E-value of 0.0 and a percent identity greater than 98% (45, 46).

### Screening for DENV and CHIKV

Since arbovirus infection can influence the mosquito microbiota, all samples were tested for DENV and CHIKV, which were the only arboviruses reported in the department of Boyacá during the year preceding sample collection (47).

A multiplex PCR was performed on cDNA from all field-caught and laboratory-adapted insects to detect DENV serotypes (DENV-1 to −4), following the protocol described by Chien et al. (48) (Table 1). The primers used were Forward mD1 (5′-TCAATATGCTGAAACGCGAGAGAAACCG-3′), Reverse rTS1 (3′-CCCGTAACACTTTGATCGCT-5′), Reverse mTS2 (3′-CGCCACAAGGGCCATGAACAGTTT-5′), Reverse TS3 (3′-TAACATCATCATGAGACAGAGC-5′), and Reverse rTS4 (3′-TTCTCCCGTTCAGGATGTTC-5′). Each reaction mixture included GoTaq Green Master Mix (2×), 10 μmol of each primer, 2.45 µL of nuclease-free water, and 1 µL of cDNA. Synthetic controls of the NSP1 gene (TWIST Q-392917), with an initial concentration of 1000 ng/mL, were used and diluted 1/1,000 to reach 1 ng/mL. The amplification program consisted of an initial activation of the DNA polymerase at 95°C for 5 min, followed by 35 cycles of denaturation at 95°C for 30 s, annealing at 57°C for 45 s, extension at 72°C for 33 s, and a final extension at 72°C for 5 min.

**TABLE 1:** List of primers used for the detection of DENV and CHIKV arboviruses

Arbovirus detection (DENV-CHIKV)					
Virus	Primer	Sequence (5′−3′)	Genomic position	Amplicon size (bp)	Amplified genomic region
DENV-1	DENV1-F	mD1-TCAATATGCTGAAACGCGAGAGAAACCG	134–322	208	C-prM
	DENV1-R	rTS1-AGCGATCAAAGTGTTACGGG			
DENV-2	DENV2-F	mD1-TCAATATGCTGAAACGCGAGAGAAACCG	134–232	119	
	DENV2-R	mTS2-AAACTGTTCATGGCCCTTGTGGCG			
DENV-3	DENV3-F	mD1-TCAATATGCTGAAACGCGAGAGAAACCG	134–400	288	
	DENV3-R	TS3-GCTCTGTCTCATGATGATGTTA			
DENV-4	DENV4-F	mD1-TCAATATGCTGAAACGCGAGAGAAACCG	134–374	260	
	DENV4-R	rTS4-GAACATCCTGAACGGGAGAA			
CHIKV	CHIKV-F	RAAGGAGTGCCGGAARGACAT	1261–1458	197	nsP1
	CHIKV-R	CTGTGGTCGTCCGGGTTGTC			

Amplicons were visualized by electrophoresis on a 2% agarose gel stained with SYBR Safe. Expected band sizes were approximately 208 bp (DENV-1), 119 bp (DENV-2), 288 bp (DENV-3), and 260 bp (DENV-4) (48).

CHIKV detection was carried out using a conventional PCR targeting the nsP1 (non-structural protein 1) gene (49). The primers used were CHIKV-F (5′-RAAGGAGTGCCGGAARGACAT-3′) and CHIKV-R (3′-GACAACCCGGACGACCACAG-5′) (Table 1). Each reaction mixture contained GoTaq Green Master Mix (2×), 10 μmol of each primer, 4.25 µL of nuclease-free water, and 1 µL of cDNA. A synthetic control (TWIST Q-397281), diluted to 1 ng/mL, was used to validate the assay. The amplification program included an initial activation at 95°C for 5 min; 35 cycles of denaturation at 95°C for 30 s, annealing at 55°C for 60 s, and extension at 72°C for 33 s; followed by a final extension at 72°C for 5 min. Amplicons were visualized on a 2% agarose gel stained with SYBR Safe, with an expected band size of approximately 190 bp (43).

### Sample pooling

Following species confirmation (*Ae. aegypti*) and screening for arboviruses, all DENV- and CHIKV-negative mosquitoes were selected for microbiota analysis. This screening was performed because arbovirus infection is known to alter microbiota composition (28, 29, 31, 50–52). For each of the four municipalities, three replicate pools of field-collected mosquitoes were prepared (29), with five individuals per pool (for a total of 15 insects per municipality). A parallel pooling strategy was applied to the laboratory-adapted mosquitoes from each corresponding municipality. In addition, three replicate pools (five individuals each) were prepared for the Rockefeller reference strain, for a total of 135 insects distributed across three groups.

Each pool was constructed by combining 2 µL of cDNA from each of the five selected insects. This pooling strategy provides a representative profile of diversity comparable to that obtained through individual-based analyses, while maximizing the information retrieved, reducing biological variability among specimens, and representing a cost-effective alternative (53–55).

### Amplification of 16S and 18S rRNA genes for microbiota profiling

From each sample pool, the full-length 16S rRNA gene was amplified to identify bacteria and archaea using primers 27F (5´-AGAGTTTGATCCTGGCTCAG-3′) and 1492R (5´-GGTTACCTTGTTACGACTT-3′), as described by Marsay et al. (56). PCR reactions were prepared using LongAmp Taq 2× Master Mix (New England Biolabs, M0287L), 10 μmol of each primer, 2.25 µL of nuclease-free water (MP Biomedicals, 2450204), and 1 µL of pooled cDNA. The thermal cycling profile consisted of an initial activation at 94°C for 30 s, followed by 30 cycles of denaturation at 94°C for 30 s, annealing at 47.9°C for 60 s, and extension at 65°C for 1 min 15 s, with a final extension at 65°C for 10 min.

To identify microeukaryotes, the hypervariable V4–V5 regions of the 18S rRNA gene were amplified using primers 566F (5′-CAGCAGCCGCGGTAATTCC-3′) and 1289R (5′-ACTAAGAACGGCCATGCACC-3′), following the protocols of Marsay et al. (56) and Hadziavdic et al. (57). The V4 region is widely recognized as the most informative subunit of the 18S gene for distinguishing microeukaryotic communities, as it exhibits the greatest sequence heterogeneity and an optimal length for taxonomic discrimination (57). By focusing sequencing on the V4–V5 segment, greater accuracy in species assignment is ensured, as the most robust reference databases, such as PR2 (Protist Ribosomal Reference database), offer exceptional coverage and curation for these specific regions (58). Reactions were prepared using the same components as those used for the 16S rRNA gene. The thermal profile included an initial activation at 94°C for 30 s; 30 cycles of denaturation at 94°C for 30 s, annealing at 54.6°C for 60 s, and extension at 65°C for 30 s; and a final extension at 65°C for 10 min.

For both amplicons, product integrity was verified by electrophoresis on a 2% agarose gel stained with SYBR Safe (Invitrogen, S33102), confirming bands of approximately 1,500 bp (16S rRNA) and 700 bp (18S rRNA).

### Library preparation and MinION sequencing

Double-stranded DNA was quantified using a Qubit fluorometer (Invitrogen), and all samples were normalized to a final concentration of 11 ng/µL. Sequencing libraries were prepared for the Oxford Nanopore MinION platform according to the manufacturer’s instructions. Briefly, DNA was end-repaired and dA-tailed using the NEBNext Ultra II End Repair/dA-Tailing Module (NEB, cat. #E7546), followed by ligation of sequencing adapters. The DNA was then purified using AMPure XP beads (AXP). Subsequently, 11 ng of DNA from each sample was ligated with native barcodes (NB01–NB96) using the Blunt/TA Ligase Master Mix. The final barcoded libraries were loaded onto R10 version flow cells (FLO-MIN114) and sequenced on a MinION device for 48 h, controlled by MinKNOW software (v.22.10.7).

### Bioinformatic and statistical analysis

#### Data processing and quality control

Basecalling and demultiplexing of the raw POD5 files were performed using Dorado v.0.9.5 (59). The initial quality of the resulting reads was assessed with NanoPlot v.1.44.1 (60). Reads were then filtered using Filtlong v.0.2.1 (GitHub - rrwick/Filtlong: quality filtering tool for long reads), retaining sequences with lengths between 1,000–2,000 bp for 16S rRNA and 500–900 bp for 18S rRNA. Post-filtering quality was re-evaluated with NanoPlot, confirming that all samples achieved mean Phred scores above 18.

#### Taxonomic assignment and abundance estimation

Taxonomic assignment and relative abundance estimation were performed using the EMU v.3.5.1 pipeline (61) with default parameters and its built-in database for 16S rRNA analyses (v.3.4.5), which, as of November 2025, contains 49,301 sequences from 17,555 unique bacterial and archaeal species (61); and using the Protist Ribosomal Reference (PR2) database v.5.1.0 (58), which, as of November 2025, contains more than 220,000 microeukaryotic sequences for 18S rRNA analyses, also with default parameters. For bacterial communities, assignments were performed at the phylum, family, genus, and species levels. Abundance plots were generated in R v.4.4.3 (RStudio 2024.12.1-563) using the packages readxl, ggplot2, dplyr, and tidyr.

To characterize the bacterial microbiota, visualizations focused on the 10 most abundant taxa at the species/genus and family levels, and on the five most abundant taxa at the phylum level, as these consistently represented more than 60% of the total sequences. Differential abundance analysis between field-collected and laboratory-reared groups was performed using ANCOM-BC2 (with the ANCOMBC and tidyverse R packages). To ensure the reliability of the results, we applied a species-level abundance filtering threshold of 0.01%. This cutoff was chosen based on the established detection limit of the EMU algorithm (61), thereby minimizing the inclusion of low-confidence taxa and potential false positives that fall below the algorithm’s sensitivity range, while controlling the false discovery rate (62).

Shared and unique species (abundance >2%) among the field, laboratory, and Rockefeller groups were identified and visualized using Venn diagrams (ggvenn, ggplot2) (63, 64). Microorganisms shared by the majority of samples (≥90% of samples) (64–68) are hypothetically proposed as members of the core microbiota, as they are present in field insects, laboratory-adapted insects, and have also been previously reported by other authors in different developmental stages and mosquito species. A heatmap of the shared microorganisms was generated using the pheatmap package, with read counts normalized by z-score transformation (scale function).

Due to the low number of organisms detected in the microeukaryotic communities, their analysis was descriptive. Abundances were averaged across the three replicates of each group (field, laboratory, and Rockefeller), focusing on microorganisms with abundances greater than 1%.

#### Diversity analysis

Bacterial alpha diversity (within-sample) was assessed using the Shannon-Weaver (H´) and Simpson (D1) indices (69, 70) via the diversity function in the R package vegan. Species richness was evaluated as the number of observed species using the specnumber function. Data distribution was tested with the Shapiro-Wilk test.

Statistical comparisons for non-normally distributed data (Shannon, Simpson) among the three main groups (field, laboratory, Rockefeller) were performed using the Kruskal-Wallis test followed by Dunn’s *post hoc* test if *P* < 0.05. For normally distributed data (richness), ANOVA followed by Tukey’s *post hoc* test was used. Pairwise comparisons between two groups were conducted using the Mann-Whitney U-test. Visualizations were created as box-and-whisker plots using vegan and ggplot2.

Beta diversity (between-sample community comparison) was evaluated using the Bray-Curtis dissimilarity index and visualized with principal coordinates analysis (PCoA), generated with the vegdist and capscale functions from vegan. Significant differences in community structure between groups were tested using a permutational multivariate analysis of variance (PERMANOVA) with 999 permutations (adonis2 function), applying a Benjamini-Hochberg correction to the *P*-values. Results were considered statistically significant at *P* < 0.05 (33, 71).

## RESULTS

### Bacterial and microeukaryotic communities differ among field-caught, laboratory-adapted, and Rockefeller strain mosquitoes

Oxford Nanopore MinION sequencing generated, on average, 12,097 raw reads per sample for the 16S rRNA gene and 63,270 raw reads per sample for the 18S rRNA gene. After length filtering, an average of 8,108 reads per sample was retained for the 16S rRNA gene, corresponding to 322 species, with a mean read length of 1,491 bp and an average quality score of 19. For the 18S rRNA gene, an average of 41,825 reads per sample was obtained; after removing sequences corresponding to the mosquito or to organisms other than fungi or parasites, seven eukaryotic microorganisms were identified, with a mean read length of 767 bp and an average quality score of 18.

**Fig 2:**
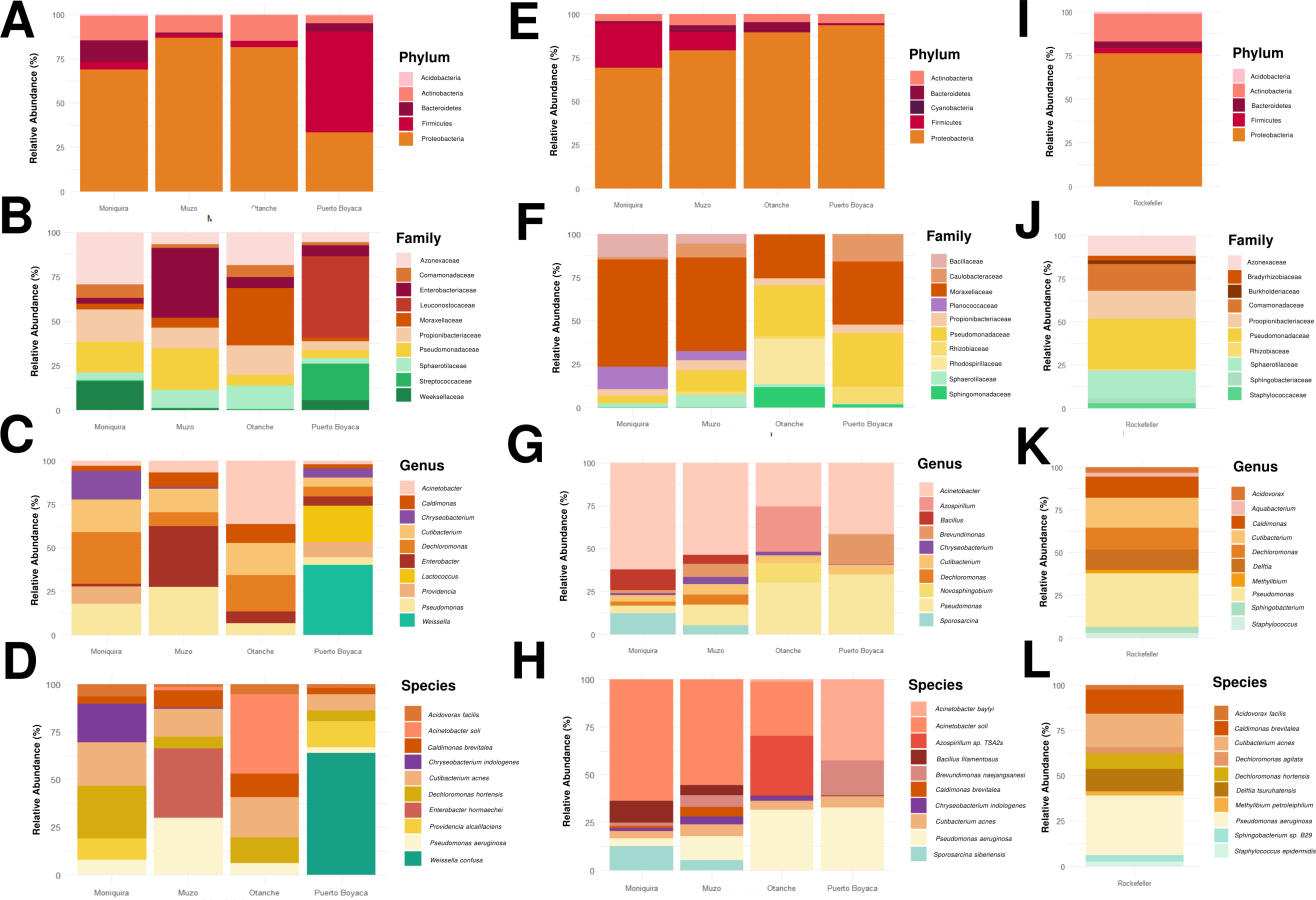
Relative abundance of the most prevalent bacterial taxa in *Ae. aegypti* from the three experimental groups. The plots display the taxonomic composition for field-caught mosquitoes ([**A**] phylum, [**B**] family, [**C**] genus, [**D**] species), laboratory-adapted populations ([**E**] phylum, [**F**] family, [**G**] genus, [**H**] species), and the Rockefeller strain ([**I**] phylum, [**J**] family, [**K**] genus, [**L**] species).

Following the confirmation that all mosquito samples were *Ae. aegypti* and tested negative for the arboviruses DENV and CHIKV, we proceeded to characterize the relative abundances of the bacterial communities present in the field-caught, laboratory-adapted, and Rockefeller strain groups. Figure 2 summarizes the taxonomic composition at multiple levels, highlighting the five most abundant phyla, as well as the 10 most abundant families, genera, and species for each condition.

At the phylum level, Proteobacteria was the most dominant taxon across all conditions. It accounted for a mean relative abundance of 67% in field-caught mosquitoes, increasing to 82.7% in laboratory-adapted populations and 76.1% in the Rockefeller strain. The phyla Actinobacteria, Firmicutes, and Bacteroidetes were shared across all three groups. In contrast, Acidobacteria was detected at low abundance (0.9%) in field and Rockefeller mosquitoes but was absent in the laboratory group, whereas Cyanobacteria was among the top 5 most abundant phyla only in laboratory-adapted insects, albeit at a very low abundance (0.2%).

At the family level, Pseudomonadaceae, Propionibacteriaceae, and Sphaerotilaceae were present in all three groups. Pseudomonadaceae showed a progressive increase in abundance from field (12.6%) to laboratory (19.1%) and Rockefeller (29.0%) mosquitoes. Conversely, Propionibacteriaceae and Sphaerotilaceae were more abundant in field (12.9% and 7.7%, respectively) and Rockefeller (16.1% and 15.5%) groups compared to the laboratory-adapted populations (4.7% and 2.8%). Several families were shared between specific pairs of groups: Moraxellaceae was abundant in both field (10.9%) and laboratory (44.4%) mosquitoes; Azonexaceae (15.0% and 11.9%) and Comamonadaceae (4.6% and 15.5%) were shared between field and Rockefeller groups; and Rhizobiaceae and Sphingomonadaceae were shared between laboratory and Rockefeller mosquitoes at lower abundances. Distinct family profiles were also observed. The field-caught group was uniquely characterized by higher abundances of Enterobacteriaceae, Leuconostocaceae, Streptococcaceae, and Weeksellaceae. In contrast, the laboratory group exclusively featured Bacillaceae, Caulobacteraceae, Planococcaceae, and Rhodospirillaceae, while the Rockefeller colony was distinguished by Bradyrhizobiaceae, Burkholderiaceae, and Staphylococcaceae.

At a finer taxonomic resolution, the genera *Cutibacterium, Dechloromonas,* and *Pseudomonas* were shared across all conditions. *Pseudomonas* demonstrated a clear gradient, with its relative abundance increasing from 14.0% in field mosquitoes to 20.3% in laboratory and 31.4% in Rockefeller specimens. *Cutibacterium* was most abundant in the Rockefeller (17.4%) and field (14.0%) groups, with lower levels in the laboratory-adapted mosquitoes (4.8%). *Dechloromonas* followed a similar pattern. The most notable genera shared only between field and laboratory populations were *Acinetobacter* (12.0% and 45.7%, respectively) and *Chryseobacterium* (5.6% and 1.9%). *Caldimonas* was shared between field (6.1%) and Rockefeller (12.3%) groups. Each condition also harbored a unique set of highly abundant genera. In field mosquitoes, these included *Enterobacter, Lactococcus, Providencia,* and *Weissella*. The laboratory group was distinguished by *Azospirillum, Bacillus, Brevundimonas, Novosphingobium,* and *Sporosarcina*. Lastly, the Rockefeller strain exclusively featured *Acidovorax, Aquabacterium, Delftia, Methylobacterium, Sphingobacterium,* and *Staphylococcus*.

At the species level, *Pseudomonas aeruginosa, Cutibacterium acnes,* and *Caldimonas brevitalea* were shared across all three groups. *P. aeruginosa* was the most prominent, increasing in abundance from field (11.6%) to laboratory (20.2%) and Rockefeller (32.8%) specimens. *Acinetobacter soli* (10.8% in field, 36.9% in lab) and *Chryseobacterium indologenes* (5.15% in field, 2.0% in laboratory) were the main species shared between field and laboratory mosquitoes. Among the 10 most abundant species, several were exclusive to a single condition. Field-caught mosquitoes uniquely harbored *Enterobacter hormaechei, Providencia alcalifaciens,* and *Weissella confusa*. The laboratory-adapted group was characterized by *Acinetobacter baylyi, Azospirillum* sp. TSA2s*, Bacillus filamentosus, Brevundimonas naejangsanensis,* and *Sporosarcina siberiensis*. Finally, the Rockefeller strain exclusively contained *Dechloromonas agitata, Delftia tsuruhatensis, Methylobacterium petroleiphilum, Sphingobacterium* sp. B29*,* and *Staphylococcus epidermidis*.

### Microeukaryotic community composition

Regarding the microeukaryotic communities (see Table S1 at https://zenodo.org/records/19859956), field-caught mosquitoes were overwhelmingly dominated by the parasite *Ascogregarina culicis*, with a mean relative abundance of 85.7%. In addition to *A. culicis*, field samples contained the yeasts *Starmerella bacillaris* (5.8%) and *Candida tropicalis* (3.7%), along with other *Candida* species (3.5%). In contrast, the laboratory-adapted mosquitoes displayed a different profile, characterized by filamentous fungi such as *Penicillium* spp. (29.2%) and *Phlebia livida* (4.9%), although *A. culicis* was still present at 21%. The Rockefeller strain mosquitoes were almost exclusively colonized by *Ascogregarina culicis* (97%).

### Beta diversity analysis reveals microbial community shifts despite comparable alpha diversity

Given the observed shifts in relative abundances across the different rearing conditions, we performed alpha and beta diversity analyses to quantitatively assess differences in the bacterial microbiota composition among the field-caught, laboratory-adapted, and Rockefeller strain mosquitoes. Alpha diversity was assessed using species richness, the Shannon index, and the Simpson index (see Fig. S1 at https://zenodo.org/records/19859956). Statistical comparisons among the three main groups were conducted using ANOVA for richness and the Kruskal-Wallis test for the Shannon and Simpson indices; pairwise comparisons were performed with the Mann-Whitney U-test. The analysis revealed no statistically significant differences (*P* > 0.05) in any of the alpha diversity metrics, either when comparing the three main conditions (see Fig. S1A at https://zenodo.org/records/19859956) or when performing pairwise comparisons between field-caught and laboratory-adapted populations from each specific municipality (see Fig. S1B at https://zenodo.org/records/19859956).

**Fig 3:**
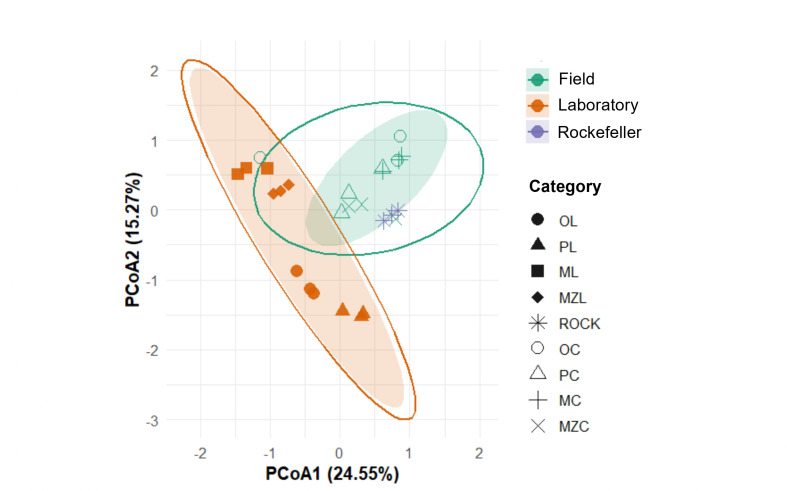
PCoA of the bacterial microbiota of *Ae. aegypti* based on Bray-Curtis dissimilarity. The plot shows the clustering of samples from field-collected populations in green: OC, PC, MC, and MZC (Otanche field, Puerto Boyacá field, Moniquirá field, and Muzo field, respectively); their laboratory-adapted counterparts in orange: OL, PL, ML, and MZL (Otanche laboratory, Puerto Boyacá laboratory, Moniquirá laboratory, and Muzo laboratory, respectively); and the Rockefeller reference strain in purple (ROCK).

In contrast, beta diversity, evaluated using Bray-Curtis dissimilarity and visualized with a PCoA, revealed significant differences in community structure among the groups (Fig. 3). A PERMANOVA test confirmed these visual patterns, showing that the microbiota composition of laboratory-adapted mosquitoes was significantly different from that of both field-caught mosquitoes (*P* = 0.003) and the Rockefeller strain (*P* = 0.01). However, no statistically significant difference was detected between the field-caught and Rockefeller strain communities.

**Fig 4:**
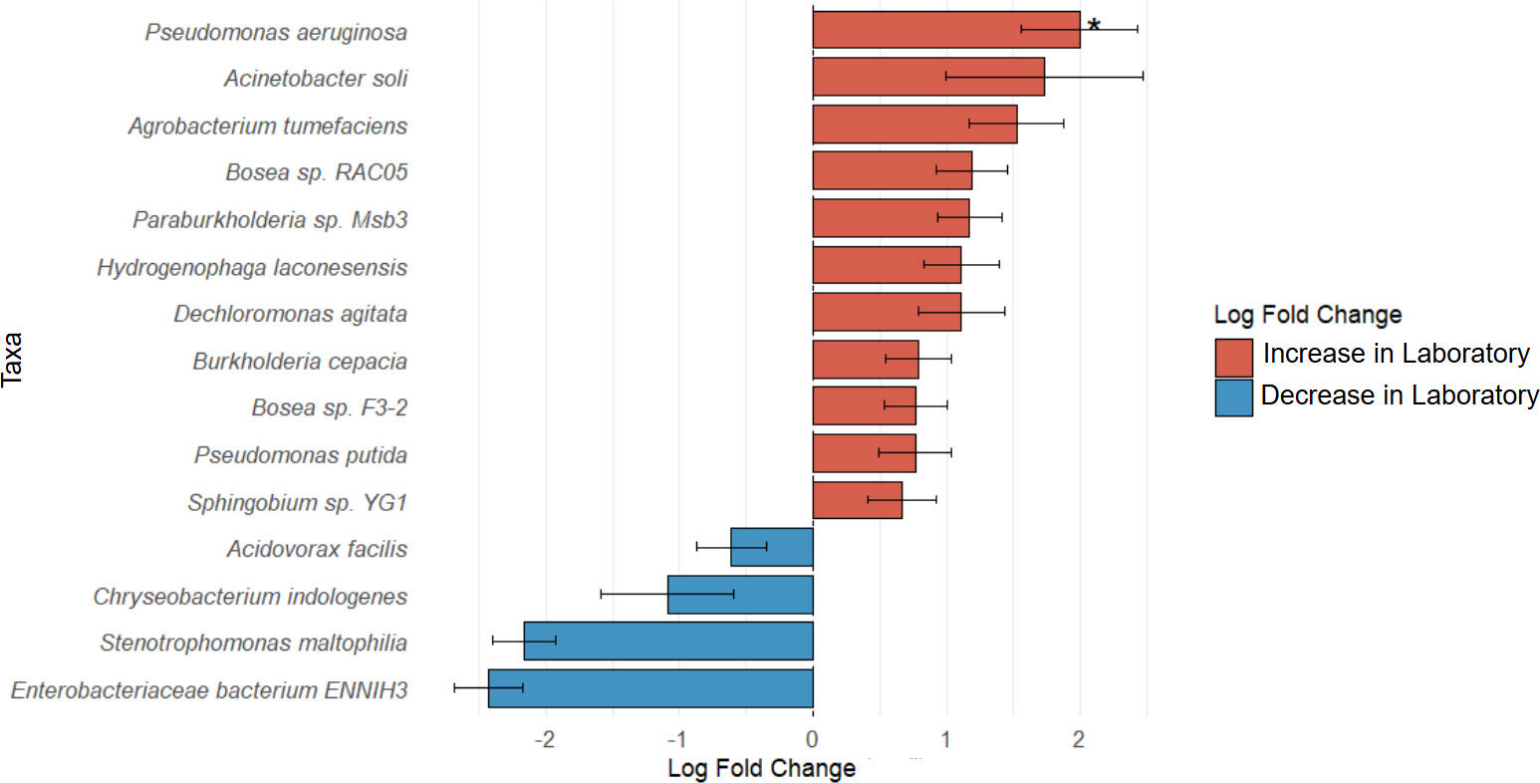
Differentially abundant taxa identified between laboratory-reared and field-caught *Ae. aegypti*. The plot illustrates microbial taxa that are significantly enriched or depleted in laboratory mosquitoes relative to their wild counterparts.

Given the absence of significant differences in alpha diversity but the clear shifts observed in beta diversity, we proceeded to identify differentially abundant species between the laboratory-adapted and field-collected groups using ANCOM-BC2 (59) (Fig. 4). The analysis indicated that several microorganisms exhibited changes in their abundances (*P* < 0.05), with some increasing and others decreasing under laboratory conditions relative to field conditions. However, *P. aeruginosa* was the only species found to be differentially abundant at a statistically significant level (*P* < 0.0001, q = 0.01). Specifically, this species was significantly enriched in laboratory-reared mosquitoes compared with their field-collected counterparts.

### Condition-specific bacteria highlight potential adaptation to rearing environments

The mosquito microbiota is shaped by the insect’s environment, including larval habitats and adult food sources, as well as by vertical transmission from females to their offspring (72). Given that this microbiota is known to influence vector immunity, reproduction, insecticide resistance, and vector competence, we sought to identify the shared bacterial members and those unique to each condition. To this end, a Venn diagram was generated to visualize unique and shared species (with a relative abundance threshold >2%) among the three experimental groups (see Fig. S2 at https://zenodo.org/records/19859956).

The analysis revealed that a substantial portion of the microbiota was condition-specific (see Fig. S2 and Table S2 at https://zenodo.org/records/19859956). Field-collected mosquitoes harbored the highest number of unique species (34 species; 51.5% of the total), followed by the laboratory-adapted group (17 species; 25.8%). The Rockefeller strain exhibited the lowest number of unique species (three species; 4.5%). A small number of species were shared between specific pairs of groups: three species (4.5%) were common to field and laboratory mosquitoes, whereas five species (7.6%) were shared between the field and Rockefeller groups. Notably, no species were shared exclusively between laboratory-adapted and Rockefeller mosquitoes. A core set of four species (6.1%) was present across all three conditions.

The species comprising this shared microbiota are detailed in Table S2 at https://zenodo.org/records/19859956. This shared microbiota, likely constituting part of the core microbiota and present regardless of rearing condition, consisted of *C. acnes*, *Dechloromonas hortensis*, *P. aeruginosa*, and *C. brevitalea*. Three additional species (*A. soli*, *C. indologenes*, and *P. putida*) were shared specifically between the field-collected and laboratory-adapted groups.

**Fig 5:**
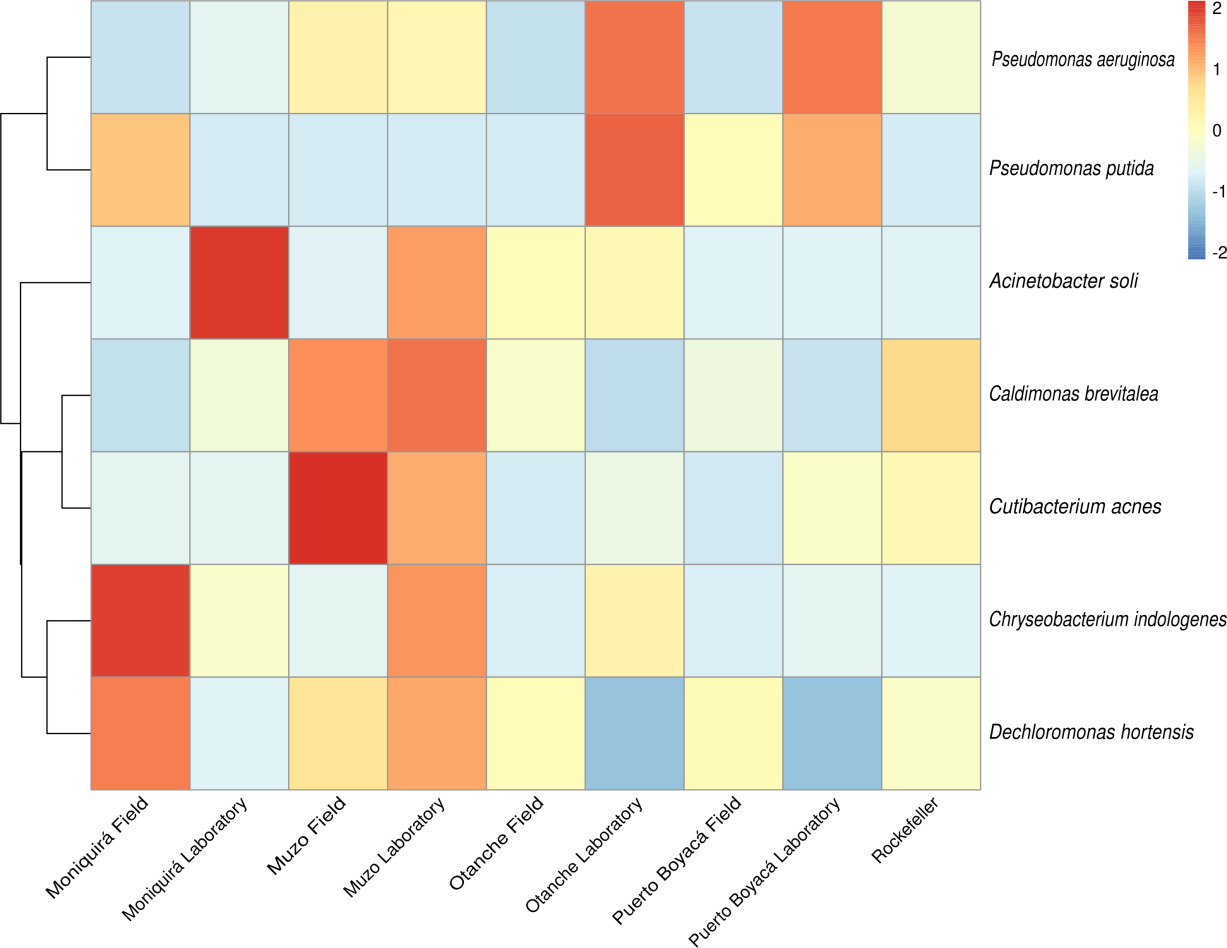
Heatmap of the seven shared bacterial species from *Ae. aegypti* based on z-score normalized read counts across field, laboratory, and Rockefeller strain samples.

To investigate whether these seven persistent taxa were maintained within specific mosquito lineages after colonization, we examined their distribution using a heatmap of z-score normalized read counts (Fig. 5). The consistent presence of certain bacteria in both field-collected populations and their corresponding laboratory-adapted populations hypothetically suggests possible transstadial transmission. For example, *D. hortensis*, *C. acnes*, *C. brevitalea*, and *P. aeruginosa* appear to be transmitted transstadially, particularly in the Muzo lineage. Similarly, *C. indologenes* was maintained in the Moniquirá lineage, *A. soli* in the Otanche lineage, and *P. putida* in the Puerto Boyacá lineage.

## DISCUSSION

Globally, both humans and animals are at risk of acquiring mosquito-borne infectious diseases (73). Beyond being mere nuisances, certain mosquito species act as vectors, transferring pathogens between hosts (74). Characterization of the *Ae. aegypti* microbiota revealed the possible presence of a core bacteriome (present in all samples [65]) at the phylum level (Fig. 2), dominated by Proteobacteria, Firmicutes, and Actinobacteria. This pattern has been previously reported in both field- and laboratory-reared mosquitoes (36, 75–77). However, microbial composition differed between conditions due to the differential representation of some of its members.

In wild populations, exclusive microorganisms of interest were identified, including *E. hormaechei* and *W. confusa* (Fig. 2D). *E. hormaechei* has been associated in the literature with the degradation of insecticides and toxins (78, 79) and with antiviral resistance against Zika and Dengue viruses through the production of metabolites (80). Previous reports of this genus in *Ae. aegypti* and *Ae. albopictus*, even in eggs, suggest transovarial transmission (81, 82). Its absence in laboratory colonies supports the hypothesis that it is a facultative symbiont, possibly acquired under environmental stress (82). In contrast, *W. confusa* was detected only in Puerto Boyacá, suggesting local acquisition. Its previously reported antimicrobial activity via bacteriocins (83) and its associations with insecticide resistance and viral modulation (63) indicate that its presence could confer a competitive advantage over other taxa (84). These findings highlight the importance of studying wild populations, in which the microbiota reflects adaptations to environmental pressures such as insecticides or arboviruses.

In laboratory-reared mosquitoes, genera such as *Brevundimonas*, *Bacillus*, *Sporosarcina*, and *Azospirillum* were detected (Fig. 2H). In particular, *B. naejangsanensis*, commonly found in purified water and capable of passing through water filters (85), could be considered a marker of the water used during rearing. *Bacillus* and *Sporosarcina*, in turn, have been reported as bacteria transported in dust particles by wind (86–89) and are characterized by remarkable environmental adaptability (88). According to our results, these taxa appear to correspond more to environmental colonizers than to natural components of the mosquito microbiota, which is consistent with previous observations in laboratory-maintained colonies (31). *A. baylyi,* also exclusive to laboratory colonies, has been associated with rearing water in *Anopheles gambiae* (90). Taken together, these observations suggest that artificial environments can introduce or favor bacteria absent from the natural microbiota, underscoring the need to monitor surfaces, food, water, and newly collected larvae in future studies.

The Rockefeller colony exhibited a distinct profile, with the exclusive presence of *D. tsuruhatensis* (Fig. 2L). This bacterium has been reported to reduce *Plasmodium* transmission in *Anopheles* (91) and to interfere with the development of *Leishmania major*, making it a promising candidate for leishmaniasis control (92). Its persistence in this long-term maintained colony suggests positive selection across generations, which warrants further investigation. Although *Delftia* has been previously reported in *Aedes* (31, 93), including associations with insecticide resistance (63), its role in arbovirus transmission remains unexplored. Therefore, further exploring its potential role in the vector competence of this important vector represents a valuable opportunity, given its demonstrated capacity to modulate the transmission of parasites of public health relevance (91, 92).

Among eukaryotes (see Table S1 at https://zenodo.org/records/19859956), *A. culicis* was detected in field-collected mosquitoes regardless of locality, representing the first report in *Ae. aegypti* from Colombia. This parasite can increase larval mortality, alter development (94, 95), and even enhance arbovirus transmission by harboring CHIKV in its oocysts (96, 97). It can also modulate bacterial colonization through mechanical or immune interactions (98). Its presence raises questions about potential effects on *Wolbachia*-based control programs, given reported correlations with *Wolbachia* in *Ae. albopictus* (98). Future studies are needed to evaluate the impact of *A. culicis* on the vector competence of *Ae. aegypti* and on CHIKV dynamics in the Colombian context. Beyond conventional viral surveillance, systematic detection of this parasite is essential to establish whether a correlation exists between *A. culicis* infection in the vector and the incidence of chikungunya cases or outbreaks in humans.

Among yeasts, *Stermerella* was detected exclusively in field-collected mosquitoes. This genus has been associated with nectar feeding and vertical transmission in bumblebees (99), with reproductive and diapause-related behaviors in females (100), and has been previously reported in wild *Ae. aegypti* (101). Therefore, its presence in field-collected mosquitoes is likely associated with nectar consumption and the frugivorous habits of these insects. As it is presumably an environmentally acquired yeast, this would explain its absence in laboratory-maintained insects, which are not exposed to these natural sources. Given that some yeasts can induce immune responses in insects (99), *Stermerella* could influence antiviral defenses against DENV. Similarly, according to the literature*, Candida* has been isolated from the midgut and ovaries of *Ae. aegypti* (14, 75, 102) and from *Culex* larvae (103), with a prevalence >70% in wild mosquitoes, suggesting acquisition from rearing water and nectar (16). Among its species, *C. tropicalis* is clinically relevant, as it accounts for a significant proportion of candidemia cases in Latin America, with mortality rates >40% and resistance to antifungals such as fluconazole (104–106). Its detection in mosquitoes warrants further studies to understand its pathogenic or symbiotic role. In laboratory colonies, filamentous fungi such as *Penicillium griseofulvum* and *Phlebia* were detected. *P. griseofulvum* has been reported to produce griseofulvin with antimicrobial properties (107, 108), whereas *Phlebia* produces metabolites such as oosporein with insecticidal, antiviral, and antibiotic activity (109, 110). These findings suggest that fungi associated with insectaries may also influence vector competence; however, further studies are needed to validate this hypothesis.

Regarding diversity, no significant differences in alpha diversity were found between field and laboratory mosquitoes (see Fig. S1 at https://zenodo.org/records/19859956). However, wild populations exhibited more even communities, in agreement with previous reports (36, 111, 112). As proposed by Baltar et al. (33), adaptation to insectary conditions may not reduce alpha diversity, but rather restructure community composition. This was reflected in the beta diversity analysis, in which microbial communities differed significantly among field, laboratory, and Rockefeller strain mosquitoes (Fig. 3). Wild populations displayed heterogeneous communities that varied among municipalities, likely reflecting the diversity of microhabitats and resources, whereas laboratory colonies showed strong convergence, dominated by *Acinetobacter* and *Pseudomonas*, particularly *P. aeruginosa* (Fig. 4). This species, present in all groups, has been reported by other authors to colonize the gut, reproductive organs, and salivary glands, and to withstand oxidative stress derived from blood feeding (14, 81), making it a key taxon in mosquito biology.

The shared microbiota comprised seven taxa (Fig. 5; see Fig. S2 and Table S2 at https://zenodo.org/records/19859956): *A. soli*, *C. indologenes*, and *P. putida* (shared between field- and laboratory-reared insects); and *C. acnes*, *D. hortensis*, *P. aeruginosa*, and *C. brevitalea* (present in all groups). Their persistence under different conditions suggests symbiotic roles (113), likely maintained through transstadial transmission; however, this remains a hypothesis and requires experimental validation. Notably, *P. aeruginosa*, *C. acnes*, and *C. brevitalea* were consistently detected at abundances >0.3% in all samples (100%), and may constitute part of the core microbiota of the insect (64–68) (Fig. 2). In agreement with other studies, *Pseudomonas* and *Acinetobacter* emerge as central components of mosquito microbiota (29, 36, 82). *Pseudomonas* dominates in *An. atroparvus* reared in the laboratory for at least 10 generations and has been proposed for paratransgenesis (29). It exhibits transstadial transmission (33, 114), adapts to fluctuating conditions (115), inhibits viruses such as La Crosse (116, 117), and produces metabolites that stimulate oviposition (118). These attributes position it as a key taxon with potential effects on vector competence.

*Acinetobacter* has been consistently reported in different mosquito species (36, 81, 119), is transmitted transstadially, and colonizes the gut, reproductive organs, and salivary glands (14, 82, 119). It contributes to blood digestion, nectar assimilation, and persistence in breeding sites (119). *A. soli* is among the dominant bacteria in *An. sinensis* (76), although its role remains unclear, justifying further studies on its influence on mosquito biology and vector competence.

*Dechloromonas* has been documented in adults of *An. coluzzii* in Africa (120) and in breeding sites and immature stages of *Ae. aegypti* in the Colombian Amazon (121, 122). The species *D. hortensis* has been reported to reduce chlorate and perchlorate and to degrade xenobiotics in aquatic environments (123, 124), which could facilitate larval development in chlorinated waters (121). In this context, this microorganism may be particularly relevant during the immature stages of the insect, which depend closely on aquatic environments for development and survival and could potentially be transferred from these habitats to the adult stage. Nevertheless, this hypothesis must be experimentally validated in future studies.

*Cutibacterium*, common on human skin (120, 125), has also been detected in the gut, ovaries, and salivary glands of *Anopheles* mosquitoes (126, 127) as part of bacterial interaction networks in the microbiota of *Aedes albopictus* fed on rabbit blood (128); and among the most abundant bacterial genera in *Anopheles nivipes* in Thailand (52). In other insects, such as *Bactrocera oleae*, it metabolizes lactate and produces propionate, suggesting a role in digestion (129). It has also been reported in eggs and nymphs of *Bemisia tabaci*, although its biological function is not yet well characterized and requires further investigation (130). Its higher abundance in larva-positive breeding sites in Kenya suggests that it may influence oviposition and larval development (131). In this context, further research is needed to clarify the functional role of this bacterium in insects, as its biological relevance remains poorly understood.

*Chryseobacterium* has been reported in *Ae. aegypti, Cx. quinquefasciatus, Ae. albopictus,* and other insect species, under both field and laboratory conditions, where it can promote axenic larval development through growth-regulating molecules (36, 112, 121, 132–134). However, some studies indicate that not all axenic mosquitoes require live bacteria to complete their development to adulthood, as this process can be replaced by a diet based on yeast extract and high concentrations of liver, suggesting that the primary role of the microbiota may be mainly nutritional (135, 136). In addition, *Chryseobacterium* can also negatively affect the development and egg production of *Ae. albopictus* (128). Its detection across all life stages of *Ae. aegypti* suggests possible vertical transmission (121, 133). Notably, according to a previous study, its abundance is higher in *Ae. aegypti* mosquitoes not infected with CHIKV than in infected ones (137); however, studies correlating the presence or abundance of this bacterium with the vector competence of the insect are still lacking. *C. indologenes*, mainly associated with aquatic and plant environments, has been reported in different mosquito species and life stages, indicating possible transstadial transmission (138–141), and may be related to insect development and growth; nevertheless, this hypothesis must be experimentally confirmed in the future, as its function may vary depending on the insect species in which it occurs (128).

In contrast, *Caldimonas* has only a few reports, mainly in *Ae. albopictus* larvae and in groundwater (142). These thermophilic and thermotolerant bacteria (143) include *C. brevitalea*, which has been reported to produce glidobactins with antifungal and antitumor properties (144, 145). Although they are rarely documented in insects, their antimicrobial potential could influence microbial interactions and, consequently, vector competence, an aspect that requires further investigation.

Overall, the shared microbiota identified in the present study may play important roles in mosquito biology. Given their persistence under both field and laboratory conditions, these bacteria could hypothetically be maintained through transstadial transmission, making them promising candidates for paratransgenesis strategies due to their multigenerational stability (93, 146). However, the enrichment of taxa such as *A. soli* and *P. aeruginosa* in laboratory-reared insects could influence viral replication and interactions with symbionts such as *Wolbachia*. Both genera have been mainly associated with *Ae. aegypti* populations free of *Wolbachia* (20). This is relevant because *Wolbachia* transinfection in *Ae. aegypti* is performed under laboratory conditions (24, 147), and the enrichment of certain microorganisms from artificial environments could affect the stability of the infection and, consequently, vector competence (23). Given that *Wolbachia* interacts with the microbiota in reproductive tissues and in the gut, such interactions could directly influence its efficacy (20, 23). Future studies should explicitly evaluate how laboratory conditions shape the microbiota of *Wolbachia*-transinfected mosquitoes to determine whether artificially acquired microorganisms affect *Wolbachia* function after release.

Finally, several limitations should be considered when interpreting our results. The number of samples analyzed was limited, and the non-probabilistic sampling performed during the dry season did not allow us to capture the influence of other environmental factors. In addition, although most laboratory-adapted insects corresponded to the 5th generation (F5), those from Moniquirá belonged to the 13th generation (F13). Although no statistically significant differences were detected in alpha and beta diversity indices between F5 and F13 insects (data not shown), the lack of generational homogeneity represents a limitation of the study. Therefore, the results should be interpreted with caution and cannot be generalized to the entire region. Future studies should include insects collected at different times of the year, a larger number of biological replicates, and ideally a longitudinal design spanning from F1 to beyond F13.

Additionally, although pooling provides a representative diversity profile comparable to that obtained from individual analyses and, by increasing total biomass, can minimize the influence of contamination, maximize information yield, reduce biological variability among specimens, and represent a cost-effective alternative (72), this approach entails important interpretative limitations. In particular, pooling prevents the assessment of interindividual variation and hampers the analysis of microbial interactions at the individual mosquito level (72, 148). It may also affect alpha and beta diversity estimates and lead to an overestimation of the core microbiota (i.e., microorganisms present in all samples) (65). Moreover, the use of three pools per condition (*n* = 3) reduces the statistical power of the tests (149, 150), limiting the ability to detect true differences (e.g., Moniquirá field vs Moniquirá laboratory). For this reason, most statistical analyses were performed by grouping all field insects (*n* = 12) and all laboratory-adapted insects (*n* = 12). Consequently, this design does not allow exploration of intrapopulation heterogeneity or the establishment of fine-scale host-microbiota associations at the individual level, and this limitation should be taken into account when interpreting the results.

Furthermore, the microbiota was analyzed from whole mosquitoes in order to provide an integrative overview (31, 81, 119); however, future studies could focus on specific organs to better understand microbial roles in vector biology. Complementary analyses of rearing water and larval food would also help clarify whether the communities observed in laboratory-adapted insects derive from transstadial transmission or were acquired under laboratory conditions. It should be noted that this descriptive study considered only prokaryotes and microeukaryotes, leaving aside other relevant microorganisms such as viruses (the virome), which can strongly influence insect microbial composition, as well as vector biology and competence (75, 151–154). Therefore, future research should include these components and evaluate their dynamics under different rearing conditions and their interactions with other microorganisms.

Likewise, it is important to emphasize that the proposed functions of the identified microorganisms are, for now, purely speculative and based on previous reports by other authors, and thus require experimental validation. In this regard, the use of metagenomic and metatranscriptomic approaches could help elucidate the functional interactions between the microbiota and the host.

Despite these limitations, our study provides valuable information on the microbial diversity of *Ae. aegypti*. Moreover, it constitutes the first report in Colombia of microeukaryotic microorganisms in *Ae. aegypti* mosquitoes, such as *Ascogregarina culicis*, highlighting the importance of studying field insects and their potential impact on public health. Overall, our results provide evidence of how environmental factors and rearing conditions shape microbial composition, laying the groundwork for exploring the role of these microorganisms in vector competence. Future studies should deepen the understanding of these interactions across generations and under different ecological contexts, particularly in the presence of arboviruses or symbionts such as *Wolbachia*, with the aim of developing more effective and sustainable control strategies.

### Conclusions

This study demonstrates that both environmental conditions and laboratory adaptation shape the microbiota composition of *Ae. aegypti*, as reflected by differences in beta diversity and by the presence of exclusive microorganisms between field and laboratory populations. Among the taxa of particular interest, *E. hormaechei*, *W. confusa*, and *A. culicis* were identified, the latter being reported for the first time in Colombia. Although its role in the transmission of arboviruses such as CHIKV remains unknown, its detection is of great interest given its potential relevance to public health. Likewise, the higher abundance of *A. soli* and *P. aeruginosa* in laboratory colonies highlights the need to consider these microorganisms in studies of virus-vector interactions and in the design of *Wolbachia*-based control strategies. Finally, the detection of a shared bacteriome suggests the existence of possible transstadial transmission processes, with important implications for the development of paratransgenesis-based vector control approaches; however, this hypothesis must be experimentally validated in future studies.

## Data Availability

The sequencing data generated in this study have been deposited in the National Center for Biotechnology Information (NCBI) under BioProject accession number PRJNA1415332 (ID 1415332). Raw sequencing reads and associated metadata are available through the NCBI Sequence Read Archive (SRA). All data are publicly accessible and comply with NCBI data-sharing policies.
